# Bayesian Meta-Analysis for Binary Data and Prior Distribution on Models

**DOI:** 10.3390/ijerph18020809

**Published:** 2021-01-19

**Authors:** Miguel-Angel Negrín-Hernández, María Martel-Escobar, Francisco-José Vázquez-Polo

**Affiliations:** Department of Quantitative Methods & TiDES Institute, University of Las Palmas de Gran Canaria, E-35017 Las Palmas de Gran Canaria, Spain; maria.martel@ulpgc.es (M.M.-E.); francisco.vazquezpolo@ulpgc.es (F.-J.V.-P.)

**Keywords:** bayesian meta-analysis, clustering, binary data, priors, frequentist validation

## Abstract

In meta-analysis, the structure of the between-sample heterogeneity plays a crucial role in estimating the meta-parameter. A Bayesian meta-analysis for binary data has recently been proposed that measures this heterogeneity by clustering the samples and then determining the posterior probability of the cluster models through model selection. The meta-parameter is then estimated using Bayesian model averaging techniques. Although an objective Bayesian meta-analysis is proposed for each type of heterogeneity, we concentrate the attention of this paper on priors over the models. We consider four alternative priors which are motivated by reasonable but different assumptions. A frequentist validation with simulated data has been carried out to analyze the properties of each prior distribution for a set of different number of studies and sample sizes. The results show the importance of choosing an adequate model prior as the posterior probabilities for the models are very sensitive to it. The hierarchical Poisson prior and the hierarchical uniform prior show a good performance when the real model is the homogeneity, or when the sample sizes are high enough. However, the uniform prior can detect the true model when it is an intermediate model (neither homogeneity nor heterogeneity) even for small sample sizes and few studies. An illustrative example with real data is also given, showing the sensitivity of the estimation of the meta-parameter to the model prior.

## 1. Introduction

Meta-analysis has been widely applied in many research areas and is of particular importance in healthcare studies. When there exist different randomized controlled clinical trials (or studies) of a particular medical treatment, a meta-analysis may be conducted to determine what final conclusion can be drawn on the important question from each study, the effectiveness of the treatment.

One of the cases that has received more attention in the literature is the meta-analysis for binary data [1]. On the one hand, because it is very common for effectiveness to be measured through a binary variable according to whether or not a certain objective has been achieved (to survive, do not relapse or to reach a low viral load). Charles et al. [2] found that half of trials calculated their sample size based on a binary outcomes. On the other hand, binary outcomes have different statistical considerations to using continuous outcomes. The Bayesian random-effects model for meta-analysis given by Sutton and Abrams [3] would not be suitable for modeling binary data:

(1) $$\begin{array}{c} x_{i}∼N\left(\theta_{i},\tau_{i}\right),\;i=1,\ldots ,k\\ \theta_{i}∼N\left(\theta ,\tau \right),\\ \theta ∼[-,-]\;\tau ∼[-,-], \end{array}$$

where $x_{i}$ denotes an observed effect for each of *k* studies, θ the estimated pooled effect, and $\tau^{2}$ is an estimate of the between-study variance. For binary data, the preceding normal hierarchical model has been applied to the logit transformation $y_{i}=log\left[x_{i}/\left(n_{i}-x_{i}\right)\right]$ with the reparametrization $log[\theta_{i}/\left(1-\theta_{i}\right)]$, where $x_{i}$ denotes the number of successes at the *i*th study [4,5,6,7]. However, this normal approximation does not work properly when the samples sizes ($n_{i}$) are small or when the number of successes is zero, even if a continuity correction is applied to the original data, as it was shown by Sweeting et al. [8].

Moreno et al. [9] proposed an objective Bayesian meta-analysis model for binary data in which no continuity correction is required. The Bayesian model proposed for the study *i* is based on the binomial distribution $\left\{M_{i}:\mathrm{Bin}\left(x_{i},\theta_{i},n_{i}\right),\pi \left(\theta_{i}\right)\right\}$, and the linking distribution between the parameters of each study $\theta_{i}$ and the meta-parameter θ, $\pi (\theta_{i},\theta )$, belongs to the Fréchet class of bidimensional distributions with fixed marginals $\pi (\theta_{i})$ and π(θ). The objective Bayesian analysis assumes that these marginals are uniform priors, Unif$\left(\theta_{i}|0,1\right)$ and Unif(θ|0,1).

The model parameters will therefore be the parameters for the *k* studies, $\theta_{1},\ldots ,\theta_{k}$, and the meta-parameter θ. However, if some of the $\theta_{i}^{\prime }s$ are equal the dimension of the model would be reduced. In [10], the authors proposed to study the between-sample heterogeneity as a model selection problem, clustering the parameters $\theta_{1},\ldots ,\theta_{k}$ based on the samples $\left(x_{1},n_{1}\right),\ldots ,\left(x_{k},n_{k}\right)$. They adopted a Bayesian approach based on product partition models proposed in [11,12]. Bayesian model selection process requires the definition of a specific model prior.

In the absence of information about the models, the uniform prior is the most common prior assumed in a Bayesian model selection problem. However, this prior does not consider the structure of the cluster problem and other alternative model priors are possible such as considering the uniform distribution in each of the hierarchy levels of the clusters, or even considering the Poisson-Intrinsic prior proposed by Casella et al. [13] which penalizes the number of clusters. Although all these priors can be considered as not informative as they do not add new information to that provided by the data, the prior probabilities assigned to each partition vary. Due to the sensitivity of the estimation of the meta-parameter to the chosen cluster, we analyze in this paper the characteristics of these model priors and in which cases each one my be preferable.

A frequentist evaluation is carried out with simulated data, where different number of studies, sample sizes, and real clusters are considered. The rest of the paper is organized as follows. The binomial Bayesian model is presented in Section 2, where the Bayesian procedure for clustering the samples and the likelihood of the meta-parameter are also given. In this section, the four model priors to be compared will be presented. The simulated data and the results of the frequentist validation are described in Section 3. Section 4 provides one illustrative example with a real dataset. Finally, Section 5 summarizes the main conclusions drawn and presents some concluding remarks.

## 2. The Bayesian Binomial Model

Assume a meta-analysis involving *k* studies that provide *k* independent discrete samples which follow a binomial distribution $\left\{\mathrm{Bin}\left(x_{i}|n_{i},\theta_{i}\right),i=1,\ldots ,k\right\}$, where $\theta_{i}$ represents the treatment effectiveness, $n_{i}$ the number of patients, and $x_{i}$ the number of successful treatments, conditional on the study *i*. We assume weak prior information on the conditional treatment effectiveness $\theta_{i}$. Accordingly, the uniform prior $\mathrm{Unif}(\theta_{i}|0,1)$ is used [14,15]. The Bayesian sampling model ($M_{i}$) for i=1,…,k studies is then given by

(2) $$M_{i}:\left\{\mathrm{Bin}\left(x_{i}|n_{i},\theta_{i}\right),\;\pi \left(\theta_{i}\right)\propto 1_{\left(0,1\right)}\left(\theta_{i}\right)\right\},$$

where

(3) Bin(xi|ni,θi)=nixiθixi(1−θi)ni−xi,xi=0,1,…,ni,

and $1_{A}$ is the indicator function that takes a value of 1 to all elements of *A*, and 0 elsewhere.

The meta-model is defined by a patient in a virtual study, which is not affected by between-study variability. The variable *x* is a binary latent variable and the meta-parameter θ defines the probability of success for this virtual patient. The distribution of this meta-variable *x* is the Bernoulli meta-model Ber(x|θ), where the meta–parameter θ represents the true (unconditional) treatment effect. The objective Bayesian meta-model *M* is then given by

(4) $$M:\left\{\mathrm{Pr}\left(x|\theta \right)=\theta^{x}{\left(1-\theta \right)}^{1-x},\;\pi \left(\theta \right)=1_{\left(0,1\right)}\left(\theta \right)\right\}.$$

### 2.1. The Linking Distribution

A distribution $\pi (\theta_{i}|\theta )$ is needed to link the experimental parameters $\theta_{i}$ and the meta-parameter θ. This linking distribution should ensure there is coherence between the conditional and marginal distributions of the experimental parameters and the meta-parameter. This requires that the corresponding bivariate distribution belongs to the class of bivariate distributions with given marginals. The class of bivariate distributions solving this problem is called the Frèchet class:

(5) $$\int_{0}^{1}\pi \left(\theta_{i},\theta \right)d\theta_{i}=\pi \left(\theta \right)\;\;\mathrm{and}\;\;\int_{0}^{1}\pi \left(\theta_{i},\theta \right)d\theta =\pi \left(\theta_{i}\right).$$

Following Moreno et al. [9], a candidate $\pi (\theta_{i},\theta )$ is constructed using the intrinsic priors for model selection [16]. The conditional intrinsic linking distributions $\left\{\pi^{I}\left(\theta_{i}|\theta ,t\right),\;t=1,2,\ldots \right\}$ arises from the model comparison between the meta-model *M* and the experimental model $M_{i}$. For any positive integer *t*, the intrinsic method gives the conditional intrinsic prior as a Beta-Binomial mixture,

(6) $$\pi^{I}\left(\theta_{i}|\theta \right)=\sum_{z=0}^{t}\mathrm{Bin}\left(z|t,\theta \right)\times \mathrm{Beta}\left(\theta_{i}|z+1,t-z+1\right).$$

In general, the bivariate intrinsic prior $\pi^{I}\left(\theta_{i},\theta |t\right)$ enjoys two interesting properties. One is that it belongs to the Fréchet class with marginals $\pi (\theta_{i})$ and π(θ) following a uniform distribution. A second one is that the concentration degree of $\pi^{I}\left(\theta_{i}|\theta ,t\right)$ around θ is controlled by the training sample size *t*, the larger the *t* the larger the concentration degree. Note that the correlation coefficient between $\theta_{i}$ and θ is ρ=t/(t+1). In practice, the hyperparameter *t* is fixed, assuming a large enough correlation between $\theta_{i}$ and θ. We assume in our examples a correlation of 0.98, which implies that t=48. Hence, for the sake of simplicity in notation, we refer to the linking distribution $\pi^{I}\left(\theta_{i}|\theta \right)$ rather than $\pi^{I}\left(\theta_{i}|\theta ,t\right)$.

As it is assumed that $\theta_{i},\;i=1,\ldots ,k$ are conditional independent given θ, the linking distribution of $\theta_{1},\ldots ,\theta_{k}$ conditional on θ is given by

(7) $$\pi^{I}\left(\theta_{1},\ldots ,\theta_{k}|\theta \right)=\prod_{i=1}^{k}\pi^{I}\left(\theta_{i}|\theta \right).$$

### 2.2. Clusters

The previous section assumes that there are *k* experimental parameters $\theta_{i},\;i=1,\ldots ,k$ to be estimated. However the dimension of the experimental model can be reduced if some of the $\theta_{i}$’s are equal. Following Moreno et al. [10], model estimation in this parametric setting is a problem of clustering the parameters $\theta_{1},\ldots ,\theta_{k}$, based on the samples $x_{1},\ldots ,x_{k}$ from the experiments. We first define what is meant by cluster. The samples $x_{i}$ and $x_{j},$i≠j, from $f(x|\theta_{i},n)$ and $f(x|\theta_{j},n),$ respectively, are said to be in the same cluster if $\theta_{i}=\theta_{j}.$ The between-sample heterogeneity is then determined by the number of clusters and by the location of the samples $\left(x_{1},n_{1}\right),\ldots ,\left(x_{k},n_{k}\right)$ within these clusters.

To cluster the samples we adopt the product partition model approach proposed by Barry and Hartigan [12], together with a Bayesian model selection procedure based on Bayes factors for the intrinsic priors for the model parameters.

**Figure 1:**
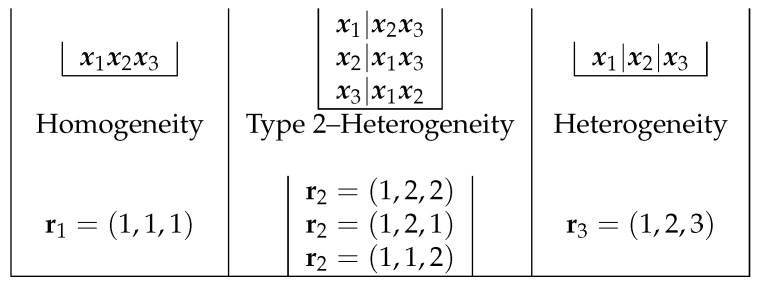
Clustering structure and different heterogeneity structures with k=3 studies.

We employ the following notations and expressions in the meta-analysis conducted [13]. For a given *p*, we define a partition of the samples into *p* clusters by the vector $r_{p}=\left(r_{1},\ldots ,r_{k}\right)$, where $r_{i},i=1,\ldots ,k$, is an integer between 1 and *p* denoting the cluster to which $x_{i}$ is assigned. Figure 1 shows the possible clustering structures for k=3, and their corresponding $r_{p}$.

### 2.3. The Likelihood of θ for a Particular Partition

The likelihood of θ will depend on the partition of the samples. Given a partition $r_{p}=\left(r_{1},\ldots ,r_{k}\right)$, the sampling distribution of $x=(x_{1},\ldots ,x_{k})$ given in (2) is

(8) f(x|p,rp,θp)=∏j=1pmjsjθjsj(1−θj)mj−sj,

where $\theta_{p}=\left(\theta_{1},\ldots ,\theta_{p}\right)$ is an unknown parameter of dimension *p*, the component $\theta_{j}$ in (8) corresponds to $r_{i}=j$, and $m_{j}=\sum_{i:r_{i}=j}n_{i}$ and $s_{j}=\sum_{i:r_{i}=j}x_{i}$ are the sample size and number of success of the cluster *j*. The likelihood of a particular partition, for example, $r_{2}=\left(1,2,2\right)$, is

f(x|2,r2=(1,2,2),θ2)=n1x1θ1x1(1−θ1)n1−x1n2+n3x2+x3θ2x2+x3(1−θ2)(n2+n3)−(x2+x3).

The heterogeneity partition $r_{k}=\left(1,2,3,\ldots ,k\right)$ has the corresponding likelihood function given by

f(x|k,rk,θk)=∏i=1knixiθixi(1−θi)ni−xi,

and the homogeneity partition $r_{1}=\left(1,1,\ldots ,1\right)$ has the corresponding likelihood function given by

f(x|1,r1,θ1)=∑i=1kni∑i=1kxiθ1∑i=1kxi(1−θ1)∑i=1k(ni−xi).

Now, integrating out $\theta_{p}$ with the intrinsic prior $\pi \left(\theta_{p}|p,r_{p}\right)=\int \pi^{I}\left(\theta_{1},\ldots ,\theta_{p}|\theta \right)1_{\left(0,1\right)}$(θ)dθ, we obtain the likelihood of θ, conditional on the cluster model $\left(p,r_{p}\right)$ given by

(9) $$\begin{array}{c} f\left(x|p,r_{p},\theta \right)=\prod_{j=1}^{p}\int_{0}^{1}f\left(x|p,r_{p},\theta_{p}\right)\pi \left(\theta_{j}|\theta \right)\;d\theta_{j}=\\ ={\left(1+t\right)}^{p}{\left(1-\theta \right)}^{tp}\prod_{j=1}^{p}\frac{\Gamma \left(s_{j}+1\right)\Gamma \left(m_{j}+t-s_{j}+1\right)}{\Gamma (m_{j}+t+2)}\left.{}_{3}F_{2}\left(a_{j},b_{j},\frac{\theta }{\theta -1}\right),\right. \end{array}$$

where $\left.{}_{3}F_{2}\left(v,w,z\right)\right.$ denotes the generalized hypergeometric function with argument *z* and vector parameters v and w of dimensions 3 and 2. In this case, the parameters $a_{j}=\left(-t,-t,s_{j}+1\right)$ and $b_{j}=\left(1,-m_{j}-t+s_{j}\right)$ are related with the number of 1’s and 0’s in cluster j, respectively.

### 2.4. The Likelihood of θ the Prior Distribution over the Partitions

To derive the likelihood function of θ we need to integrate out (9) with respect to a discrete prior on $\left(p,r_{p}\right)$. The (unconditional) likelihood of θ for the data x is given by

(10) $$f\left(x|\theta \right)=\sum_{p=1}^{k}\left(\sum_{r_{p}}f\left(x|p,r_{p},\theta \right)\pi \left(p,r_{p}|k\right)\right).$$

The prior distribution on the partitions $\pi (p,r_{p}|k)$ plays an important role in the estimation of the parameter θ [13]. We consider here four priors on $\left(p,r_{p}\right)$ which are motivated by reasonable but different assumptions. The four selected prior distribution assume the absence of prior information about the models, but ranges from the assignment of high prior probability at the boundary p=1 and p=4 (homogeneity and heterogeneity structures, respectively) to other intermediate situations that moderate the a priori assignment to these two clusters or considers them all equally probable.

**Figure 2:**
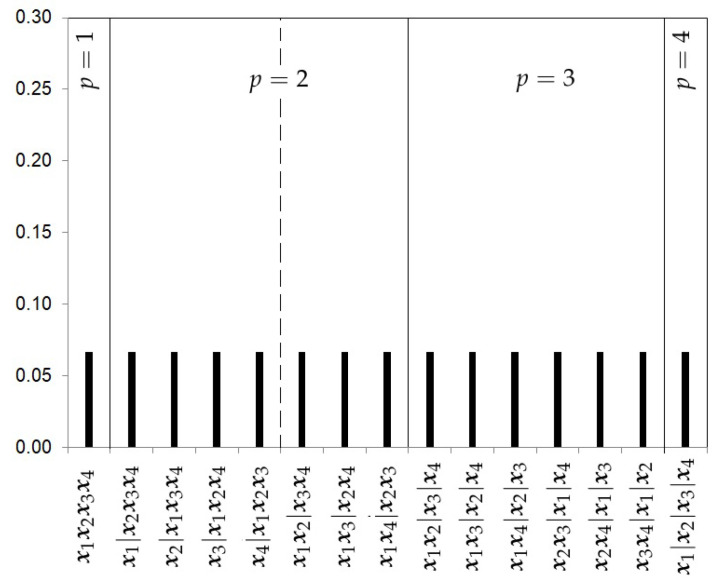
Uniform prior probabilities for the partitions with k=4 studies.

**Figure 3:**
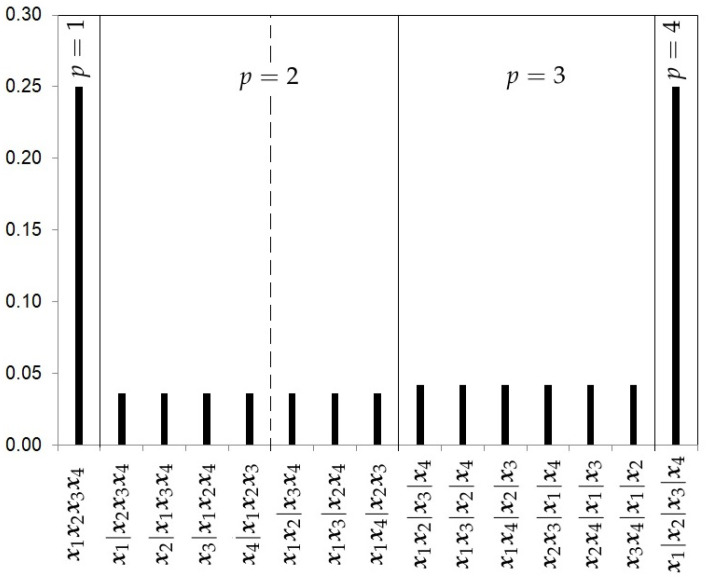
Hierarchical Uniform prior with 2 levels probabilities for the partitions with k=4 studies.

**Figure 4:**
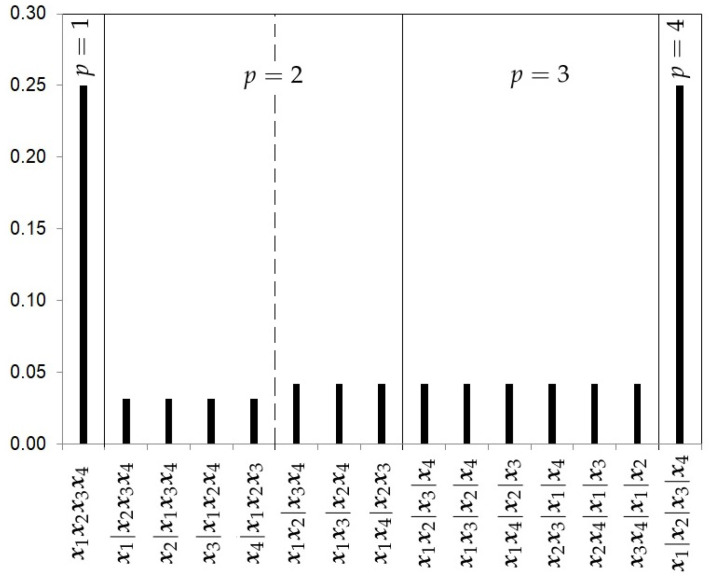
Hierarchical Uniform prior with 3 levels probabilities for the partitions with k=4 studies.

**Figure 5:**
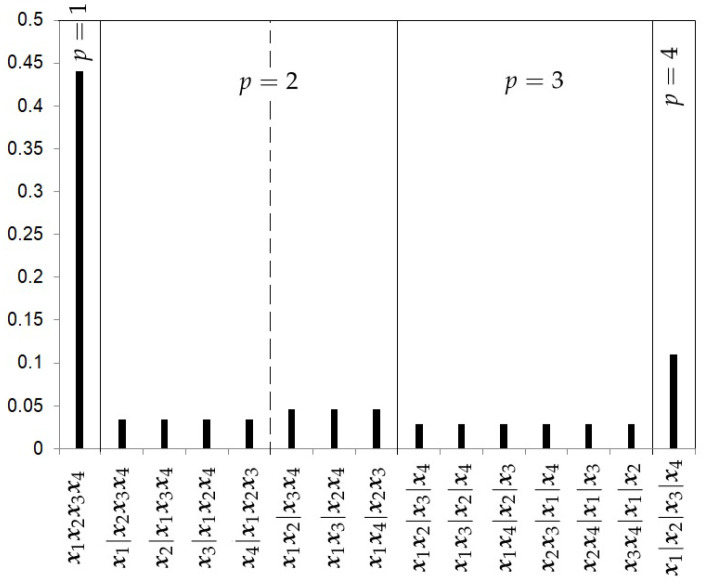
Hierarchical Poisson prior with 3 levels probabilities for the partitions with k=4 studies.

- The Uniform prior.
  The first prior proposed is the uniform prior (*U*), which gives the same probability to every model, that is,
  (11) $$\pi^{U}\left(p,r_{p}|k\right)=\frac{1}{B_{k}},$$
  where $B_{k}$, the Bell number, is the number of subsets a set of size *k* can be partitioned into. Figure 2 shows the prior probabilities for each partition when four studies are considered. In this example, the Bell number is 15. This choice does not take into account the level of complexity of each partition.
- The Hierarchical Uniform Prior with 2 levels (HU2).
  As recommended by Casella et al. [13], a hierarchical uniform prior can be appropriate to take into account the different levels of complexity of the partitions. This prior distribution distinguishes two levels of complexity in the partitions. The first level is given by the number of clusters *p* in which the *k* samples are grouped. The second level will be given by the number of possible partitions of the *k* samples into *p* clusters. Let $R_{p}$ represent this set of partitions into *p* clusters, which we call the cluster class. The number of partitions in $R_{p}$ is given by the Stirling number of the second kind S(k,p) and can be written as
  (12) S(k,p)=∑1≤k1≤…≤kpk1+…+kpk1⋯kp1R(k1,…,kp),
  where k1+…+kpk1⋯kp is the multinomial coefficient and $R\left(k_{1},\ldots ,k_{p}\right)=\prod_{i=1}^{k}\left[\sum_{j=1}^{p}I\left(k_{j}=i\right)\right]!$ corrects the count by considering the redundant strings corresponding to the vector $\left(k_{1},\ldots ,k_{p}\right)$. For instance, to calculate the Stirling number S(4,2), there are two possible vectors $\left(k_{1},k_{2}\right)$, the vector (1,3), and the vector (2,2), and the Stirling number would be
  (13) S(4,2)=41,31R(1,3)+42,21R(2,2)=4!1!3!11!1!+4!2!2!12!=4+3=7,
  which is the number of possible partitions for p=2 and k=4.
  The hierarchical uniform distribution for 2 levels will be given by the decomposition
  (14) $$\pi^{HU2}\left(p,r_{p}|k\right)=\pi \left(r_{p}|p,k\right)\pi \left(p|k\right)=\frac{1}{S(k,p)}\frac{1}{k}.$$
  Figure 3 shows the prior probabilities for each partition using the hierarchical uniform prior with 2 levels with 4 studies. Note that this hierarchical distribution assigns a higher prior probability to cases of homogeneity and heterogeneity.
- The Hierarchical Uniform Prior with 3 levels (HU3).
  Following Casella et al. [13] and Moreno et al. [10], the prior specification for $\left(p,r_{p}\right)$ can be decomposed in three levels:
  (15) $$\pi^{HU3}\left(p,r_{p}|k\right)=\pi \left(p,r_{p}|R_{p;k_{1},\ldots ,k_{p}},k\right)\pi \left(R_{p;k_{1},\ldots ,k_{p}}|p,k\right)\pi \left(p|k\right).$$
  Unlike the previous prior distribution, the hierarchical uniform prior with 3 levels considers the number of ways the integer *k* can be partitioned into *p* clusters. We will call it the number of configuration classes within each $R_{p}$ and it will be denoted by b(k,p). In our illustrative example with k=4, this value is equal to 1 for p=1,3,4 (b(4,1)=b(4,3)=b(4,4)=1), and only for the cluster class p=2 there are two configuration classes, corresponding to the configurations x|xxx and xx|xx, so b(4,2)=2.
  The hierarchical uniform prior with 3 levels is given by the expression
  (16) $$\pi^{HU3}\left(p,r_{p}|k\right)=\frac{k_{1}!\cdot \ldots \cdot k_{p}!}{k!}\frac{R(k_{1},\ldots ,k_{p})}{b(k,p)}\frac{1}{k}.$$
  Figure 4 shows the prior probabilities for each partition using the hierarchical uniform prior with 3 levels and 4 studies.
- The Hierarchical Poisson Prior with 3 levels (HP3).
  Casella et al. [13] argue that when analyzing a cluster problem of a sample size *k*, the extreme case of having *k* clusters should be given *a priori* a smaller probability than that given to any other case. Extending this argument for any *k*, it might be reasonable that the prior distribution on the number of clusters π(p|k) might be a truncated Poisson distribution P(p|λ), where λ is an unknown parameter. We can assume an intrinsic prior $\pi^{I}\left(\lambda |\lambda_{0}=1\right)$ for λ, constructed by testing the Poisson null hypothesis $H_{0}:\lambda =\lambda_{0}$ versus $H_{1}:\lambda \in R^{+}$ [17],
  (17) $$\pi^{I}\left(\lambda |\lambda_{0}=1\right)=\frac{\lambda^{-1/2}}{\Gamma (1/2)}e^{-(\lambda +1)}\left.{}_{0}F_{1}\left(1/2,\lambda \right)\right.,$$
  where ${}_{0}F_{1}\left(1/2,\lambda \right)$ denotes the confluent hypergeometric function. The reason for taking $\lambda_{0}=1$ is that the one cluster model is the reference model throughout the analysis. The resulting marginal intrinsic distribution for *p* is
  (18) $$\pi^{I}\left(p|k\right)=\frac{m^{I}\left(p\right)}{\sum_{p=1}^{k}m^{I}\left(p\right)},p=1,\ldots ,k,\;m^{I}\left(p\right)=\int_{0}^{+\infty }\frac{\lambda^{p}e^{-\lambda }}{p!}\pi^{I}\left(\lambda |\lambda_{0}=1\right)d\lambda .$$
  We cannot assume a Poisson distribution for the other two levels of the hierarchical structure because there is no a clear order in relation to complexity. For this reason, a uniform distribution is assumed for the other two levels. The hierarchical Poisson prior will be given by
  (19) $$\pi^{HP3}\left(p,r_{p}|k\right)=\frac{k_{1}!\cdot \ldots \cdot k_{p}!}{k!}\frac{R(k_{1},\ldots ,k_{p})}{b(k,p)}\pi^{I}\left(p|k\right).$$
  Figure 5 shows the prior probabilities for each partition using the hierarchical Poisson prior with 4 studies. The prior probability for the homogeneity cluster is more than four times higher than the prior probability of the heterogeneity case.

Finally, from (10) and the priors defined in (11), (14), (16) and (19), the (unconditional) likelihood of θ for the data x is given by

(20) $$f\left(x|\theta \right)=\sum_{p=1}^{k}\left(\sum_{r_{p}}f\left(x|p,r_{p},\theta \right)\pi \left(p,r_{p}\right)\right).$$

### 2.5. Bayesian Model Averaging in the Meta-Analysis

The BMA approach to meta-analysis involves averaging over all the possible models (heterogeneity structures or partitions) when making inferences about the parameter of interest θ.

In this case, the posterior probabilities correspond to those of any heterogeneity structure given by a pair $\left(p,r_{p}\right)$, which is represented by

(21) $$\mathrm{Pr}\left(p,r_{p}|x,k\right)=\frac{m_{r_{p}}\left(x|p,r_{p}\right)\pi \left(p,r_{p}\right)|k}{\sum_{p=1}^{k}\left(\sum_{r_{p}}m_{r_{p}}\left(x|p,r_{p}\right)\pi \left(p,r_{p}|k\right)\right)},$$

where $m_{r_{p}}\left(x|p,r_{p}\right)=\int f\left(x|p,r_{p},\theta_{p}\right)\pi \left(\theta_{p}|p,r_{p}\right)\;d\theta_{p}$ is the marginal of the data x conditional on model $(p,r_{p}),$ with $f(x|p,r_{p},\theta_{p})$ and $\pi (\theta_{p}|p,r_{p})$. These posterior model probabilities $\mathrm{Pr}(p,r_{p}|x)$ are the weights for the meta inference.

The posterior distribution for the parameter of interest θ becomes

(22) $$\pi \left(\theta |x\right)=\sum_{r_{p}}\pi \left(\theta |x,p,r_{p}\right)\mathrm{Pr}\left(p,r_{p}|x,k\right),$$

where

(23) $$\pi \left(\theta |x,p,r_{p}\right)=\frac{f(x|p,r_{p},\theta )}{\int_{0}^{1}f\left(x|p,r_{p},\theta \right)\;d\theta },\;0<\theta <1.$$

The posterior distribution in (22) is computed numerically using Wolfram Mathematica (see code in the supplementary material Section).

## 3. Simulated Data and Frequentist Validation

### 3.1. Simulated Data

This section presents the simulated data used in the frequentist validation. The data have been simulated from binomial distributions, where the number of studies included in the meta-analysis (*k*), the partition for the data ($r_{p}$), and the sample size within each study (*n*) vary between simulations.

The values for the number of studies (*k*) in the meta-analysis are 3, 5. Other greater values of *k* are obviously possible. For instance, we developed the case k=8 (see supplementary material Section) where the conclusions obtained are similar to that in the cases k=3 and 5. Therefore, in order to facilitate the reading of the Table 1 we only present the cases k=3 and k=5. These numbers of studies are manageable to do this simulation exercise. In this respect, Davey et al. [18] conducted an extensive review of the Cochrane Database of Systematic Reviews (CDSR) and pointed out that just under 75% of the meta-analyses contained five or fewer studies.

The sample size within each study is also a crucial parameter of the simulation. For simplicity we assume a common sample size for the *k* studies and this sample size takes values of 10, 30, 100, and 300. Finally different “true” partitions are considered for each *k*, where the heterogeneity and homogeneity cases are always included and one or two intermediate cases are also analyzed. Table 1 shows the parameters of the simulated data.

The $\theta_{i}$ parameters used in the simulation are sufficiently disparate between clusters to expect that with moderate sample sizes, the Bayesian selection process will be able to detect the true model. For all simulation scenarios, 500 simulations were performed. To analyze the properties of the prior distributions over posterior probabilities of the partitions we show the proportion of times the true model is found as the model with the highest posterior probability and the mean posterior probability in those cases in which the true model is found as the most probable. We also show the number of cases the homogeneity cluster ($r_{1}=\left(1,1,\ldots ,1\right)$) and the heterogeneity case ($r_{k}=\left(1,2,3,\ldots ,k\right)$) are found as the most probable model.

### 3.2. Frequentist Evaluation

**Figure 6:**
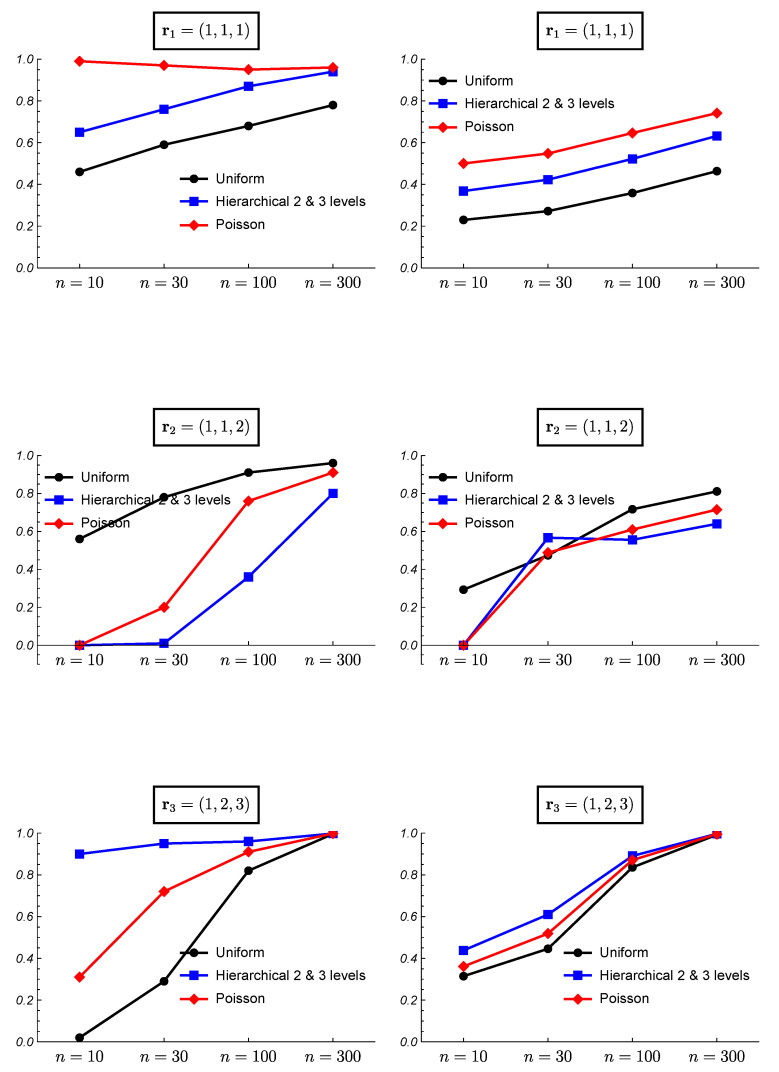
Frequentist validation for the case k=3 and true partitions $r_{1}=\left(1,1,1\right),r_{2}=\left(1,1,2\right)$ and $r_{3}=\left(1,2,3\right).$ (**Left column**) proportion of times the true partition is found as the most probable. (**Right column**) mean of the posterior probability for the true partition when it is found as the most probable.

**Figure 7:**
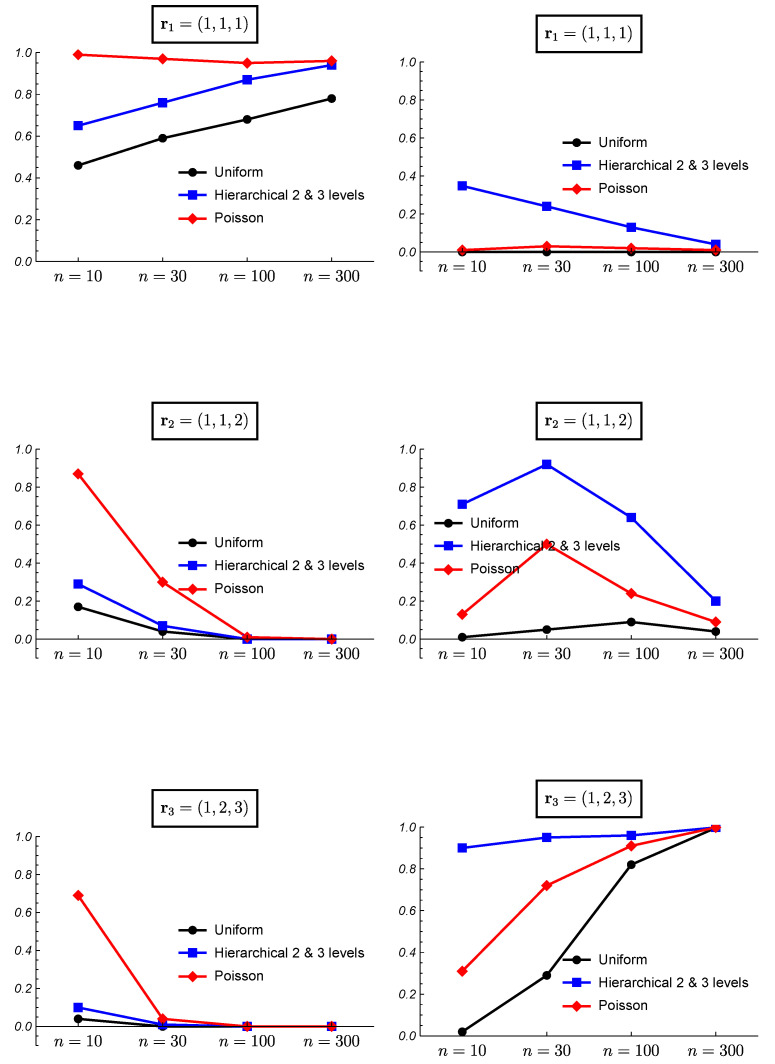
Frequentist validation for the case k=3 and true partitions $r_{1}=\left(1,1,1\right),r_{2}=\left(1,1,2\right)$ and $r_{3}=\left(1,2,3\right).$ (**Left column**) proportion of times the homogeneity case is found as the most probable. (**Right column**) proportion of times the heterogeneity case is found as the most probable.

Figure 6 and Figure 7 show the results of the frequentist validation for the case k=3 and true partitions $r_{1}=\left(1,1,1\right),r_{2}=\left(1,1,2\right)$ and $r_{3}=\left(1,2,3\right)$ corresponding to a situation of homogeneity, intermediate heterogeneity, and heterogeneity, respectively. As expected, for the true case of homogeneity $r_{1}$, the uniform prior shows worse performance as it is the only one that does not assume an a priori preference for the homogeneity. However, the results show how the uniform prior reaches a proportion of correct choices close to 70% with sample sizes greater than 100. The mean posterior probabilities reach values greater than 30% and 40% for sample sizes of 100 and 300, respectively. Observe that b(3,p)=1,1≤p≤3, thus the results from HU2 and HU3 priors are identical. The hierarchical Poisson prior, which assigns a higher prior probability to the homogeneity case, reaches a proportion of correct choices higher than 95% even with sample sizes of 10, and posterior probabilities higher than 50%. All the prior distributions show a good performance for high sample sizes.

When the true partition is an intermediate one, the results vary. Figure 6 shows the results for the true case $r_{2}=\left(1,1,2\right)$. The uniform prior shows the best performance although the proportion of right choices is smaller than 50% for n=10. The mean posterior probability reaches a value of 80% with a sample size of 300. With the hierarchical Uniform priors the true model is never chosen as the most probable for a sample size of n=10. In this case, the mean of the posterior probability for the true partition when it is found as the most probable does not exist and it is shown as 0 in the figure. For small sample sizes these prior distributions found the heterogeneity case as the most probable (Figure 7). The hierarchical Uniform priors only achieve the 50% of right choices for a sample size of 300. The hierarchical Poisson prior improves the behavior of the hierarchical Uniform priors although for a small sample size of n=10, it chooses the homogeneity case more than 85% of the simulations (Figure 7).

When the true model is the heterogeneity case, i.e., $r_{3}=\left(1,2,3\right)$, the hierarchical Uniform priors (HU2 and HU3) show the best performance even with small sample sizes. Surprisingly, the uniform prior shows worse results than those of the hierarchical Poisson prior. This can be explained by the higher prior probabilities assigned to intermediate partitions by the uniform prior, which hinder the identification of the heterogeneity case as the true model. The proportion of right choices and the mean posterior probabilities for the true model are near to 100% for all the prior models when the sample size is 300.

**Figure 8:**
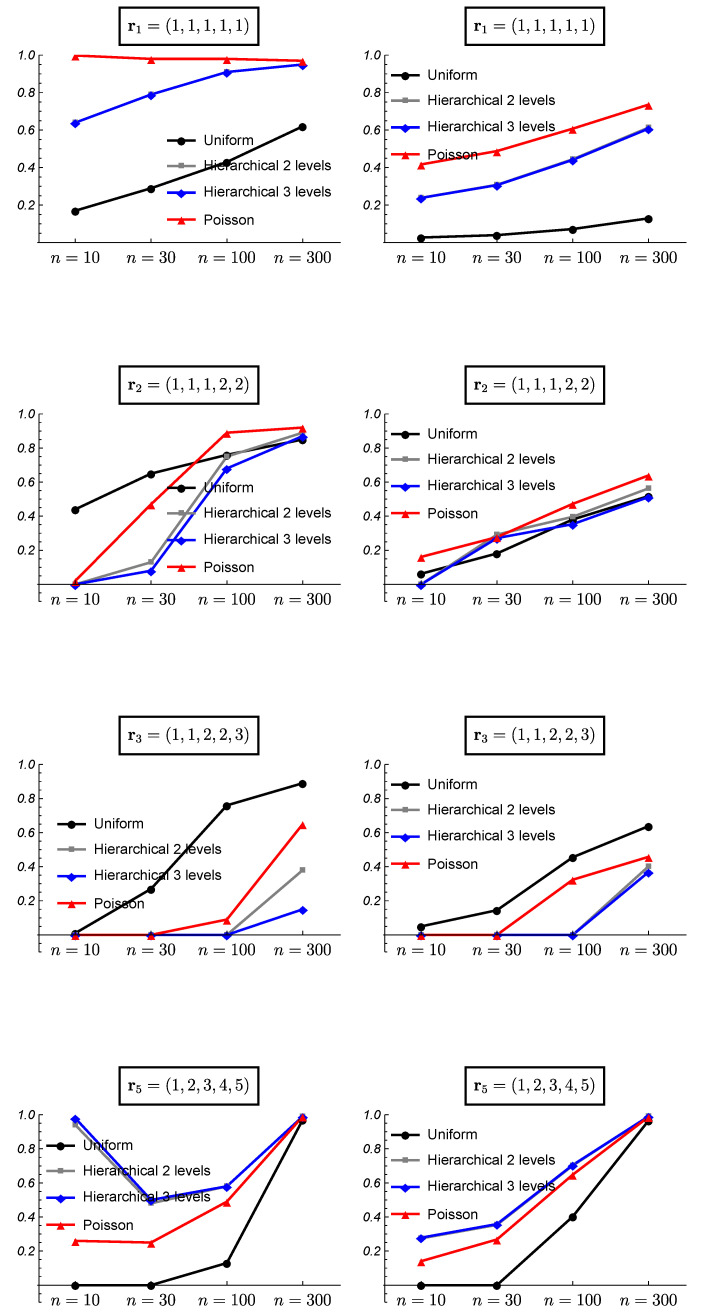
Frequentist validation for the case *k* = 5 and true partitions $r_{1}=\left(1,1,1,1,1\right),r_{2}=\left(1,1,1,2,2\right),r_{3}=\left(1,1,2,2,3\right)$, and $r_{5}=\left(1,2,3,4,5\right)$. (**Left column**) proportion of times the true partition is found as the most probable. (**Right column**) mean of the posterior probability for the true partition when it is found as the most probable.

**Figure 9:**
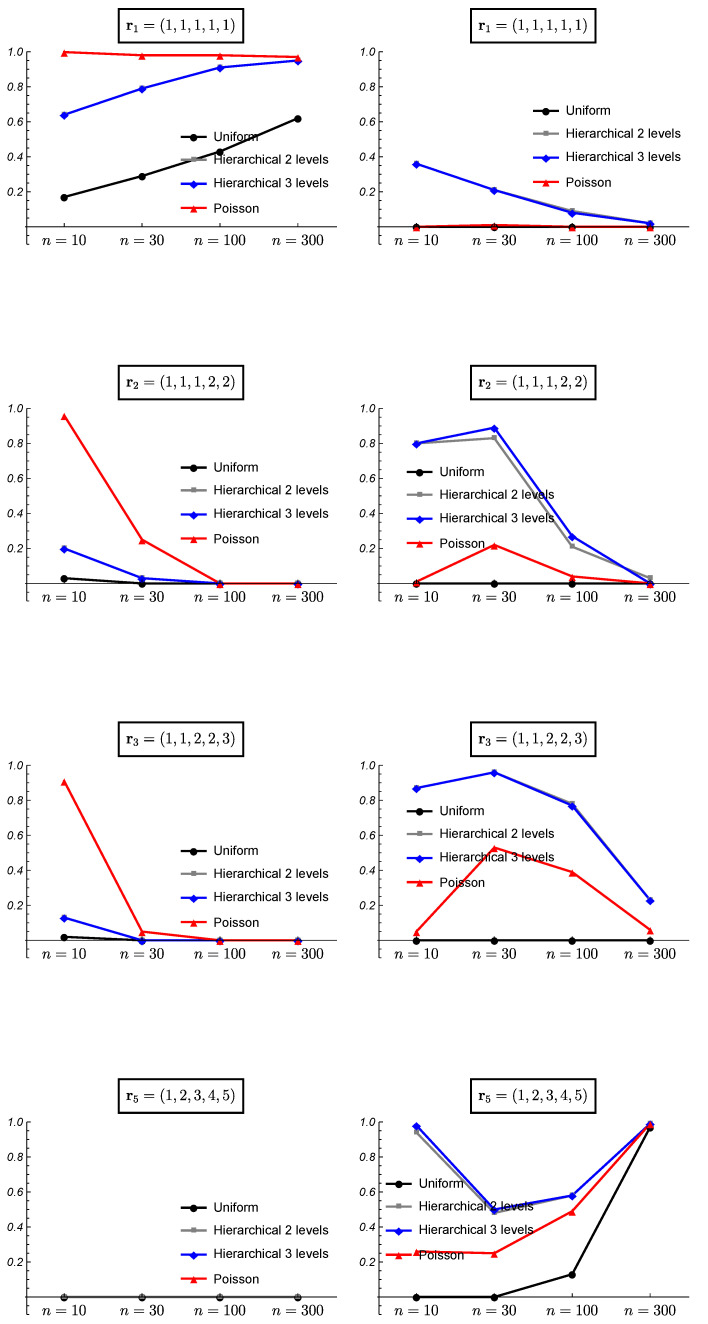
Frequentist validation for the case *k* = 5 and true partitions $r_{1}=\left(1,1,1,1,1\right),r_{2}=\left(1,1,1,2,2\right),r_{3}=\left(1,1,2,2,3\right)$, and $r_{5}=\left(1,2,3,4,5\right)$. (**Left column**) proportion of times the homogeneity case is found as the most probable. (**Right column**) proportion of times the heterogeneity case is found as the most probable.

Figure 8 and Figure 9 show the results of the frequentist validation for the case k=5 and true partitions $r_{1}=\left(1,1,1,1,1\right)$, homogeneity case, $r_{2}=\left(1,1,1,2,2\right)$ and $r_{3}=\left(1,1,2,2,3\right),$ intermediate situations and $r_{5}=\left(1,2,3,4,5\right)$, heterogeneity case.

The analysis of the homogeneity case with k=5 shows a bad performance of the uniform prior, even worse than observed for k=3. The proportion of right choices only exceed 50% for a sample size of 300. As it is shown in the Figure 9, the uniform prior found as the most probable model some intermediate models as the proportion of cases in which the heterogeneity case is chosen is 0. As it was found with k=3, the hierarchical Poisson prior shows a better performance than the HU2 and HU3 which becomes similar as the sample size increases. Some results obtained for the HU2 and HU3 priors are quite similar, showing an overlapping behavior in some cases.

Once again, the analysis of the intermediate cases with k=5 shows a similar behavior to that observed with k=3. The uniform prior reaches a higher proportion of correct choices, although for the case of two clusters (p=2), the hierarchical Poisson improves it for sample sizes greater than 100. With a moderate number of clusters (p=3), all the prior models show difficulties to choose the true model with small sample sizes. In the case of the hierarchical Poisson prior, it chooses the homogeneity case for small sample sizes, while the uniform prior chooses other intermediate models (Figure 9). The hierarchical uniform priors never choose the true model with sample sizes smaller than 300, showing preference for the heterogeneity case.

For the heterogeneity case $r_{5}=\left(1,2,3,4,5\right)$, the proportion of right choices show a U-shape for all model priors except the Uniform prior. For small sample sizes, the greater prior probability assigned to the extreme cases leads to a preference for the heterogeneity case (the homogeneity case is never chosen as it is shown in Figure 9). As the sample size increases, the importance of the prior information is reduced and other intermediate partitions are chosen, probably due to the small difference in the true probability of success between the 5 studies (see Table 1). Finally, with a sample size of 300, all model prior distributions choose the true model.

An additional analysis for the case k=8 is shown in the supplementary material Section. The results are very similar to those obtained for the case k=5.

## 4. An Illustrative Example with Real Data

In this section, we show an illustrative example with real data to analyze the impact of the prior models over the estimation of the meta-parameter θ. With the objective to determine the effectiveness of granulocyte transfusions compared to no granulocyte transfusions for treating infections in patients with neutropenia or disorders of neutrophil function in reducing mortality, Stanworth et al. [19] conducted a meta-analysis. The dataset in Table 2 is extracted from Stanworth et al. [19] and corresponds to the mortality analysis in four studies (k=4, subgroup analysis for studies transfusing greater than $1\times 10^{10}$ granulocytes at days 20–22) for granulocyte transfusions for treating infections in patients with neutropenia or neutrophil dysfunction treated with transfusion (Treatment).

**Figure 10:**
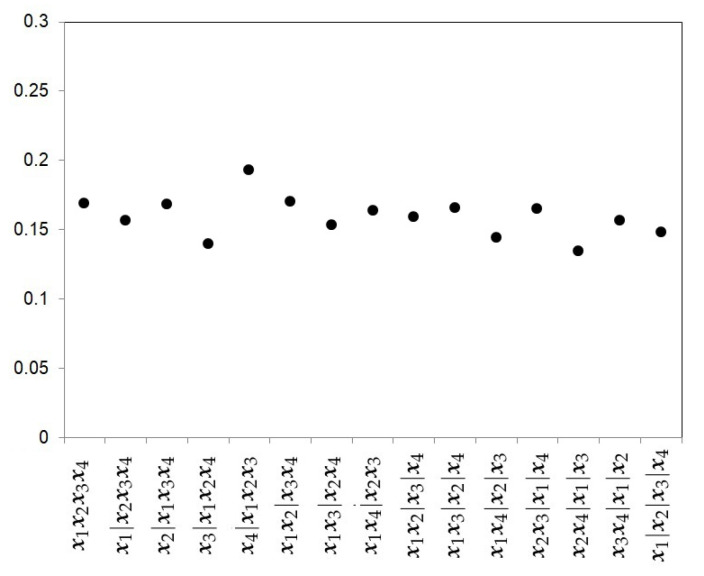
Posterior mean of the meta–parameter θ for each partition.

This is a good example to apply the model proposed by Moreno et al. [9] as the number of cases is small and there are even no cases in one study. For k=4, there are 15 possible partitions and the estimation of the meta-parameter θ will depend on the partition considered. Figure 10 shows the posterior mean conditioned on each partition. The posterior mean varies from the 0.1929 obtained for the partition $r_{2}=\left(1,1,1,2\right)$ to 0.1344 for the partition $r_{3}=\left(1,2,3,2\right)$.

The four prior models are applied to this dataset. The prior probabilities assigned to each partition can be shown in Figure 2, Figure 3, Figure 4 and Figure 5. To include into the analysis the model uncertainty, the posterior distributions for θ are all averaged in the BMA posterior distribution, and this BMA posterior distribution depends on the model priors assumed. Table 3 shows the top cluster models for the four model priors.

Top cluster models and their posterior probabilities are sensitive to the model prior. For the uniform prior model, the most probable model is $\left\{x_{1}x_{2}x_{3}|x_{4}\right\}$, with a posterior probability of 0.19. However, this model is found as the second most probable model for the hierarchical uniform priors, and reaches the third position for the Poisson prior. These last models found the heterogeneity case as the most probable model.

Table 2 shows a different mortality rate between studies, where study 4 stands out with a mortality rate of 41.18%, while the mortality rate for the other studies do not exceed 12%. The uniform prior is the only model capable of detecting this structure as the most probable model. The other prior models discard the homogeneity case but cannot detect the intermediate model, showing preference for the heterogeneity case. In this example, the uniform prior would be the best choice.

This analysis also points out the importance of the BMA estimation as it allows the model uncertainty to be included in the estimation of the meta-parameter. As can be seen, the estimation of the meta-parameter by BMA is less sensitive to the choice of the prior distribution for the models, ranging from 0.159 for the hierarchical Uniform priors to 0.164 for the Uniform prior.

## 5. Conclusions

Bayesian methods for the design, analysis, and synthesis of clinical trials have been developed in several areas including meta-analysis where the structure of the between-sample heterogeneity is essential in estimating the meta-parameter. As part of the design of the Bayesian framework, we address the question from a different standpoint, arguing that between-sample heterogeneity is a clustering problem and that model uncertainty can be incorporated into the inference using a Bayesian procedure. Under this procedure, the posterior probabilities of the cluster models are computed and the definition of the prior distribution over the models takes on special importance.

Meta-analysis for binary data is an increasingly used tool for estimating the effectiveness of a certain treatment. Meta-analysis for binary data presents interesting statistical challenges that have been addressed in the literature, such as the presence of zeros, that make it difficult to apply logit transformations to the data [20,21]. The definition of an objective Bayesian meta-analysis for binary data that does not require transformations to the data represented an advance in literature [9].

The objectivity of this analysis is given by the prior distribution assumed for the experimental parameters ($\theta_{i}$) and the meta-parameter (θ) [22]. However, when the between-sample heterogeneity is considered in the analysis as a problem of clustering the experimental parameters ($\theta_{i}$), the objectivity remains in doubt since the Bayesian model selection requires the definition of the prior distributions over the models. The hierarchical structure of the clusters does not allow to conclude that the Uniform prior distribution is the best or unique option. Moreno et al. [10] proposed to use the hierarchical Uniform prior with three levels, but other options are possible.

In this paper we analyze the properties of four model priors assuming the absence of prior information about the cluster model: the Uniform prior, the hierarchical Uniform prior with two and three levels and the hierarchical Poisson prior. There are other priors proposed by the literature for the problem of clustering, such as the Ewens–Pitman prior [23,24,25] or the Jensen–Liu prior [26]. However, these prior distributions require the assessing of a hyperparameter that reflects the a priori information about the models.

A first conclusion achieved from the frequentist validation is that none of the prior distributions is completely non-informative. The posterior probabilities for the models are very sensitive to the model priors, even with moderately large sample sizes. A useful guideline for daily practice could be as follows. If you consider that the homogeneity case is probable, the hierarchical Poisson prior for small samples sizes, or the hierarchical Uniform prior for moderately large sample sizes are the best options. If you consider that the heterogeneity case is probable, the hierarchical Uniform priors are preferable. Finally, if you consider that the real cluster is not the homogeneity or heterogeneity cases, the Uniform prior can be used for small number of studies and sample sizes.

A second conclusion is that carrying out a meta-analysis based on a single partition (even if it is the partition with the maximum posterior probability) can obtain biased results as it ignores a very important part of the uncertainty around the estimation of the meta-parameter. The BMA procedure offers a natural way to incorporate this model uncertainty into the estimation of θ [27].

As the BMA procedure implies estimating the meta-parameter for all possible partitions, computational difficulties arise when the number of studies *k* is moderately large due to time required for estimation. In that case, a set of good cluster models can certainly by found using a stochastic algorithm [13].

## Figures and Tables

**Table 1:** Parameters for the simulation data.

True Model ($r_{p}$)	Parameters ($\theta_{i}$’s)	Sample Sizes ($n_{i}$)
k=3		
1,1,1	(0.5,0.5,0.5)	(10,30,100,300)
1,1,2	(0.5,0.5,0.2)	(10,30,100,300)
1,2,3	(0.7,0.5,0.2)	(10,30,100,300)
k=5		
1,1,1,1,1	(0.5,0.5,0.5,0.5,0.5)	(10,30,100,300)
1,1,1,2,2	(0.5,0.5,0.5,0.2,0.2)	(10,30,100,300)
1,1,2,2,3	(0.7,0.7,0.5,0.5,0.2)	(10,30,100,300)
1,2,3,4,5	(0.9,0.7,0.5,0.3,0.1)	(10,30,100,300)

**Table 2:** Data in Stanworth et al. [19].

Study	Treatment	
	Events	Total
Herzig 1977 ($x_{1}$)	1	16
Higby 1975 ($x_{2}$)	2	17
Scali 1978 ($x_{3}$)	0	13
Vogler 1977 ($x_{4}$)	7	17

**Table 3:** Top cluster models in Stanworth et al. for treatment data.

Prior # 1: Uniform		Prior # 2: HU with 2 levels		Prior # 3: HU with 3 Levels		Prior # 4: HP with 3 Levels	
Cluster Model	Post. Prob.	Cluster Model	Post. Prob.	Cluster Model	Post. Prob.	Cluster Model	Post. Prob.
$x_{1}x_{2}x_{3}|x_{4}$	0.19	$x_{1}|x_{2}\left|x_{3}\right|x_{4}$	0.37	$x_{1}|x_{2}\left|x_{3}\right|x_{4}$	0.37	$x_{1}|x_{2}\left|x_{3}\right|x_{4}$	0.21
$x_{1}x_{3}\left|x_{2}\right|x_{4}$	0.14	$x_{1}x_{2}x_{3}|x_{4}$	0.11	$x_{1}x_{2}x_{3}|x_{4}$	0.10	$x_{1}x_{2}x_{3}x_{4}$	0.18
$x_{1}x_{2}\left|x_{3}\right|x_{4}$	0.11	$x_{1}x_{3}\left|x_{2}\right|x_{4}$	0.09	$x_{1}x_{3}\left|x_{2}\right|x_{4}$	0.09	$x_{1}x_{2}x_{3}|x_{4}$	0.14
$x_{1}\left|x_{2}x_{3}\right|x_{4}$	0.11	$x_{1}x_{2}x_{3}x_{4}$	0.08	$x_{1}x_{2}x_{3}x_{4}$	0.08	$x_{1}x_{3}\left|x_{2}\right|x_{4}$	0.8
$x_{1}|x_{2}\left|x_{3}\right|x_{4}$	0.09	$x_{1}x_{2}\left|x_{3}\right|x_{4}$	0.08	$x_{1}x_{2}\left|x_{3}\right|x_{4}$	0.08	$x_{1}x_{3}|x_{2}x_{4}$	0.08
the rest	<0.09	the rest	<0.07	the rest	<0.07	the rest	<0.07
BMA estimates of the meta-parameter θ							
Posterior mean: 0.164		Posterior mean: 0.159		Posterior mean: 0.159		Posterior mean: 0.163	
95% HDI: 0.050–0.317		95% HDI: 0.049–0.312		95% HDI: 0.049–0.311		95% HDI: 0.048–0.327	

## Data Availability

Mathematica codes implementing the simulate and real data experiment are available on the supplementary material Section.

## Supplementary Materials

The following are available at https://www.mdpi.com/1660-4601/18/2/809/s1.

## References

[1] Thomas D., Radji S., Benedetti A. (2014). Systematic review of methods for individual patient data meta- analysis with binary outcomes. BMC Med. Res. Methodol..

[2] Charles P., Giraudeau B., Dechartres A., Baron G., Ravaud P. (2009). Reporting of sample size calculation in randomised controlled trials: Review. BMJ.

[3] Sutton A.J., Abrams K.R. (2001). Bayesian methods in meta–analysis and evidence synthesis. Stat. Methods Med. Res..

[4] Morris C.N., Norm S.L., Bernardo J.M., Berger J.O., Dawid A.P., Smith A.F.M. (1992). Hierarchical models for combining information and for meta–analyses. Bayesian Statistics 4.

[5] Carlin J.B. (1992). Meta–analysis for 2*x*2 tables: A Bayesian approach. Stat. Med..

[6] Sutton A.J., Abrams K.R., Jones D.R., Sheldon T.A., Song F. (2000). Methods for Meta–Analysis in Medical Research.

[7] Bhaumik D.K., Amatya A., Norm S.L.T., Greenhouse J., Kaizar E., Neelon B., Gibbons R.D. (2012). Meta–Analysis of rare binary adverse event. J. Am. Stat. Assoc..

[8] Sweeting M.J., Sutton A.J., Lambert P.C. (2004). What to add to nothing? Use and avoidance of continuity corrections in meta–analysis of sparse data. Stat. Med..

[9] Moreno E., Vázquez–Polo F.J., Negrín M.A. (2014). Objective Bayesian meta–analysis for sparse discrete data. Stat. Med..

[10] Moreno E., Vázquez–Polo F.J., Negrín M.A. (2018). Bayesian meta–analysis: The role of the between–sample heterogeneity. Stat. Methods Med. Res..

[11] Hartigan J. (1990). Partition models. Commun. Stat. Theory Met..

[12] Barry D., Hartigan J.A. (1992). Product partition models for change point problems. Ann. Stat..

[13] Casella G., Moreno E., Girón F.J. (2014). Cluster analysis, model selection, and prior distributions on models. Bayesian Anal..

[14] Tuyl F., Gerlach R., Mengersen K. (2008). A comparison of Bayes–Laplace, Jeffreys, and other priors: The case of zero events. Am. Stat..

[15] Tuyl F., Gerlach R., Mengersen K. (2009). Posterior predictive arguments in favor of the Bayes–Laplace prior as the consensus prior for binomial and multinomial parameters. Bayesian Anal..

[16] Berger J.O., Pericchi L.R. (1996). The intrinsic Bayes factor for model selection and prediction. J. Am. Stat. Assoc..

[17] Moreno E. (2005). Objective Bayesian methods for one–sided testing. Test.

[18] Davey J., Turner R.M., Clarke M.J., Higgins J.P. (2011). Characteristics of meta–analyses and their component studies in the Cochrane Database of Systematic Reviews: A cross–sectional, descriptive analysis. BMC Med. Res. Methodol..

[19] Stanworth S., Massey E., Hyde C., Brunskill S.J., Navaretter C., Lucas G., Marks D., Paulus U. (2010). Granulocyte transfusions for treating infections in patients with neutropenia or neutrophill dysfunction (Review). Cochrane Libr..

[20] Friede T., Röver C., Wandel S., Neuenschwander B. (2017). Meta–analysis of few small studies in orphan diseases. Res. Syn. Methods.

[21] Pateras K., Nikolakopoulos S., Mavridis D., Roes K.C.B. (2018). Interval estimation of the overall treatment effect in a meta–analysis of a few small studies with zero events. Contemp. Clin. Trials Comm..

[22] Berger J. (2006). The case for objective Bayesian analysis. Bayesian Anal..

[23] Crowley E.M. (1997). Product partition models for normal means. J. Am. Stat. Assoc..

[24] Quintana F.A., Iglesias P.L. (2003). Bayesian clustering and product partition models. J. R. Stat. Soc. Ser. B Stat. Methodol..

[25] McCullagh P., Yang J. (2006). Stochastic classification models. Proceedings of the International Congress of Mathematicians.

[26] Jensen S.T., Liu J.S. (2008). Bayesian Clustering of Transcription Factor Binding Motifs. J. Am. Stat. Assoc..

[27] Negrín M.A., Vázquez–Polo F.J. (2008). Incorporating model uncertainty in cost–effectiveness analysis: A Bayesian model averaging approach. J. Health Econ..

