# Supplementary material for: Bayesian Meta-Analysis for Binary Data and Prior Distribution on Models
Source: Int J Environ Res Public Health. 2021 Jan 19;18(2):809. doi: 10.3390/ijerph18020809 (PMC7832911; doi:10.3390/ijerph18020809)
Supplement: Supplementary file 1 [file ijerph-18-00809-s001.pdf]

Supplementary Materials: Bayesian meta–analysis for binary data and prior distribution on models

Simulated data and frequentist validation for case $k = 8$

Table S1. Parameters for the simulation data (case $k = 8$).

True model ($\mathbf{r}_p$)	Parameters (θ_i's)	Sample sizes (n_i)
	$k = 8$	
$\mathbf{r}_1 = (1,1,1,1,1,1,1,1)$	$(0.5,0.5,0.5,0.5,0.5,0.5,0.5,0.5)$	$(10,30,100,300)$
$\mathbf{r}_2 = (1,1,1,1,2,2,2,2)$	$(0.5,0.5,0.5,0.5,0.2,0.2,0.2,0.2)$	$(10,30,100,300)$
$\mathbf{r}_3 = (1,1,1,2,2,2,3,3)$	$(0.7,0.7,0.7,0.5,0.5,0.5,0.2,0.2)$	$(10,30,100,300)$
$\mathbf{r}_8 = (1,2,3,4,5,6,7,8)$	$(0.8,0.7,0.6,0.5,0.4,0.3,0.2,0.1)$	$(10,30,100,300)$

Publisher's Note: MDPI stays neutral with regard to jurisdictional claims in published maps and institutional affiliations.

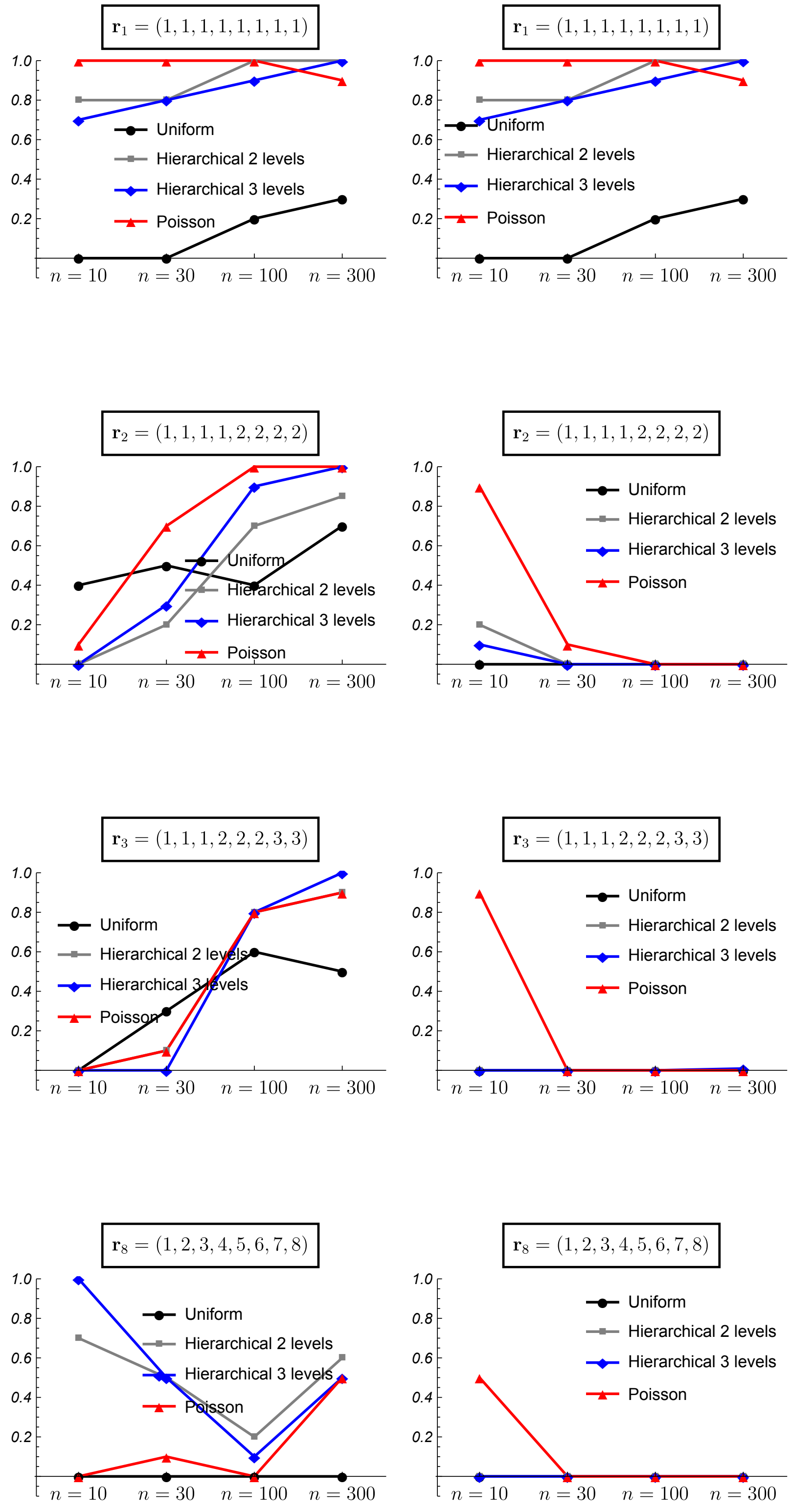

Figure S1. Frequentist validation for the case $k = 8$ and true partitions $\mathbf{r}_1 = (1,1,1,1,1,1,1,1)$, $\mathbf{r}_2 = (1,1,1,1,2,2,2,2)$, $\mathbf{r}_3 = (1,1,1,2,2,2,3,3)$ and $\mathbf{r}_8 = (1,2,3,4,5,6,7,8)$. **Left column**: proportion of times the true partition is found as the most probable. **Right column**: mean of the posterior probability for the true partition when it is found as the most probable.

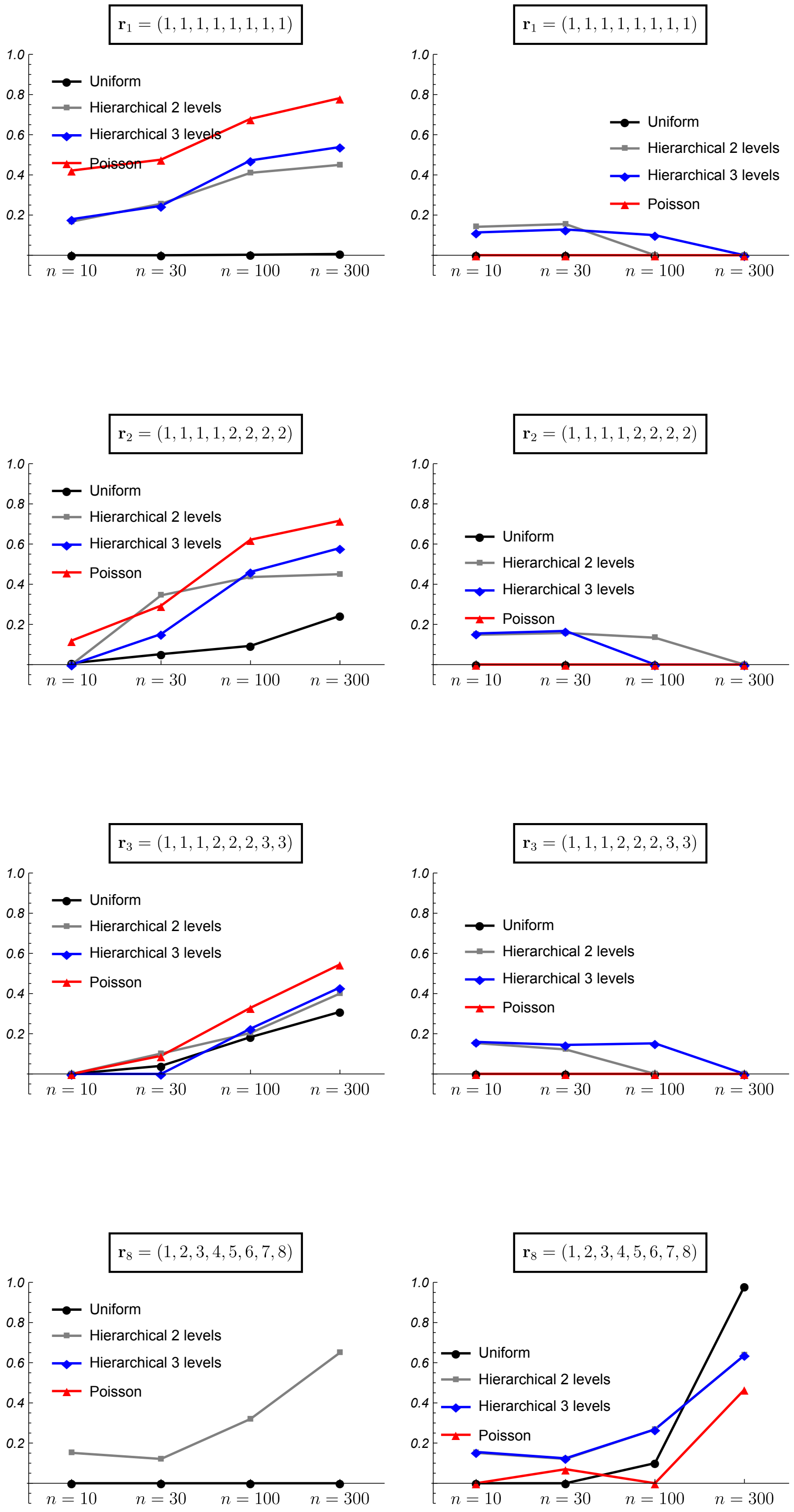

Figure S2. Frequentist validation for the case $k = 8$ and true partitions $\mathbf{r}_1 = (1,1,1,1,1,1,1,1)$, $\mathbf{r}_2 = (1,1,1,1,2,2,2,2)$, $\mathbf{r}_3 = (1,1,1,2,2,2,3,3)$ and $\mathbf{r}_8 = (1,2,3,4,5,6,7,8)$. **Left column**: proportion of times the homogeneity case is found as the most probable. **Right column**: proportion of times the heterogeneity case is found as the most probable.

Analysis with simulated data

```
(* Code for the k=3 case but it can easily be adapted to the case k=5.*)
```

Simulated data

```
n = ConstantArray[10, 3]; (*Replace 10 by 30,
100 or 300 for the other analyses*)
simulation = 500;
phi = {0.5, 0.5, 0.5}; (*Parameters of the simulation. Replace
 {0.5,0.5,0.5} by {0.5,0.5,0.2} or {0.7,0.5,0.2} for the other analyses*)
Truemodel = {{1, 2, 3}}; (*True model. Replace {{1,2,3}} by
 {{1,2},{3}} or {{1},{2},{3}} for the other analyses*)
Homogeneity = {{1, 2, 3}};
Heterogeneity = {{1}, {2}, {3}};
xvector =
  Table[Flatten[Table[RandomVariate[BinomialDistribution[n[[i]], phi[[i]]], 1],
     {i, 1, Length[phi]}]], {j, 1, simulation}];
```

Partitions

```
Needs["Combinatorica`"];

k = Length[xvector[[1]]];
models = SetPartitions[k];
Numberofmodels = Length[models];
kpartitions = KSetPartitions[k, #] & /@ Range[k];
Numbersubgroups1 = Length /@ kpartitions;
Numbersubgroups2 = Count[Length /@ Partitions[k], #] & /@ Range[k];

modelsLengths = Sort /@ Map[Length, models, {2}];

xpartitions =
  Table[Map[Total[xvector[[i]][[#]]] &, models, {2}], {i, 1, simulation}];
npartitions = Map[Total[n[[#]]] &, models, {2}];
```

Marginal distributions

Marginales;

```
F[x_, n_, t_, zeta_] :=
  (1 + t) * (Gamma[x + 1] * Gamma[n + t - x + 1] / Gamma[n + t + 2]) * HypergeometricPFQ[
    {-t, -t, 1 + x}, {1, -n - t + x}, zeta / (zeta - 1)] * (1 - zeta) ^ t;
G[x_List, n_List, t_, zeta_] := Product[F[x[[i]], n[[i]], t, zeta],
   {i, 1, Length[x]}];
Predictive[x_, n_, t_] := NIntegrate[10 ^ 1000 * G[x, n, t, zeta], {zeta, 0, 1}];
t0 = 48;
Pred[x_, n_] := Predictive[x, n, t0] / 10 ^ 1000;

Marginals = Table[Parallelize[
    MapThread[Pred, {xpartitions[[i]], npartitions}]], {i, 1, simulation}];
```

Prior distributions choose one

Uniform prior

```
Probini = ConstantArray[1 / Numberofmodels, Numberofmodels];
```

Hierarchical Uniform with 2 levels

```
Prob1Groups = Table[1 / k, {i, k}];
Prob1 = Prob1Groups[[Length /@ models]];
modelsLengths = Sort /@ Map[Length, models, {2}];
Prob2 =
  (1 / Count[Table[Length[modelsLengths[[i]]], {i, 1, Numberofmodels}], #]) & /@
   Table[Length[models[[i]]], {i, 1, Numberofmodels}];
Probini = Prob1 * Prob2 // N;
```

Hierarchical Uniform with 3 levels;

```
Prob1Groups = Table[1 / k, {i, k}];
Prob1 = Prob1Groups[[Length /@ models]];
 Prob2 = 1 / (Numbersubgroups2[[Length /@ models]]);
 modelsLengths = Sort /@ Map[Length, models, {2}];
 Prob3 = 1 / (Count[modelsLengths, #] & /@ modelsLengths);
Probini = Prob1 * Prob2 * Prob3 // N;
```

Poisson Hierarchical with 3 levels;

```
A[lambda_] :=
 lambda^(-1/2) * Exp[-(lambda + 1)] * Hypergeometric0F1[0.5, lambda] / Gamma[0.5]
B[p_] := NIntegrate[lambda^p * Exp[-lambda] * A[lambda] / Factorial[p],
   {lambda, 0, 500}];
Prob1b = Table[B[j], {j, 1, k}] / Total[Table[B[j], {j, 1, k}]];
Prob1 = Table[Prob1b[[Length[models[[i]]]]], {i, 1, Numberofmodels}];
 Prob2 = 1/(Numbersubgroups2[[Length /@ models]]);
 modelsLengths = Sort /@ Map[Length, models, {2}];
 Prob3 = 1/(Count[modelsLengths, #] & /@ modelsLengths);
Probini = Prob1 * Prob2 * Prob3 // N;
```

Posterior probabilities

```
posterior1 = Table[Probini * Marginals[[i]], {i, 1, simulation}];
posterior = Table[posterior1[[i]] / Total[posterior1[[i]]], {i, 1, simulation}];

result = Table[Partition[Riffle[posterior[[i]], models], 2], {i, 1, simulation}];
results = Table[ Sort[result[[i]], #1[[1]] > #2[[1]] &], {i, 1, simulation}];
```

Results

```
Bestmodels = Table[results[[i]][[1]][[2]], {i, 1, simulation}];

Rightdecision = N[Count[Bestmodels, Truemodel] / simulation]

PositionRight = Flatten[Position[Bestmodels, Truemodel]];

ProbabilityRight = Mean[Table[results[[i]][[1]][[1]], {i, PositionRight}]]

Homogeneitydecision = N[Count[Bestmodels, Homogeneity] / simulation]

PositionHomogeneity = Flatten[Position[Bestmodels, Homogeneity]];

ProbabilityHomogeneity =
 Mean[Table[results[[i]][[1]][[1]], {i, PositionHomogeneity}]]

Heterogeneitydecision = N[Count[Bestmodels, Heterogeneity] / simulation]

PositionHeterogeneity = Flatten[Position[Bestmodels, Heterogeneity]];

ProbabilityHeterogeneity =
 Mean[Table[results[[i]][[1]][[1]], {i, PositionHeterogeneity}]]
```

Analysis with real data

```
(* n and xvector refer to the sample
 size and the number of successes for each study,
respectively. Data for the illustrative example with k=4.*)
n = {16, 17, 13, 17};
xvector = {1, 2, 0, 7};
```

Partitions

```
Needs["Combinatorica`"];

k = Length[xvector];
models = SetPartitions[k];
Numberofmodels = Length[models];
kpartitions = KSetPartitions[k, #] & /@ Range[k];
Numbersubgroups1 = Length /@ kpartitions;
Numbersubgroups2 = Count[Length /@ Partitions[k], #] & /@ Range[k];

modelsLengths = Sort /@ Map[Length, models, {2}];

xpartitions = Map[Total[xvector[[#]]] &, models, {2}];
npartitions = Map[Total[n[[#]]] &, models, {2}];
```

Marginal distributions

```
F[x_, n_, t_, zeta_] :=
  (1 + t) * (Gamma[x + 1] * Gamma[n + t - x + 1] / Gamma[n + t + 2]) * HypergeometricPFQ[
    {-t, -t, 1 + x}, {1, -n - t + x}, zeta / (zeta - 1)] * (1 - zeta) ^t;
G[x_List, n_List, t_, zeta_] := Product[F[x[[i]], n[[i]], t, zeta],
   {i, 1, Length[x]}];
Predictive[x_, n_, t_] := NIntegrate[10^1000 * G[x, n, t, zeta], {zeta, 0, 1}];
t0 = 48;
Pred[x_, n_] := Predictive[x, n, t0] / 10^1000;

Marginals = Parallelize[MapThread[Pred, {xpartitions, npartitions}]];
```

Prior distributions choose one

Uniform prior

```
Probini = ConstantArray[1 / Numberofmodels, Numberofmodels];
```

Hierarchical Uniform with 2 levels

```
Prob1Groups = Table[1 / k, {i, k}];
Prob1 = Prob1Groups[[Length /@ models]];
modelsLengths = Sort /@ Map[Length, models, {2}];
Prob2 =
  (1 / Count[Table[Length[modelsLengths[[i]]], {i, 1, Numberofmodels}], #]) & /@
   Table[Length[models[[i]]], {i, 1, Numberofmodels}];
Probini = Prob1 * Prob2 // N;
```

Hierarchical Uniform with 3 levels;

```
Prob1Groups = Table[1 / k, {i, k}];
Prob1 = Prob1Groups[[Length /@ models]];
 Prob2 = 1 / (Numbersubgroups2[[Length /@ models]]);
 modelsLengths = Sort /@ Map[Length, models, {2}];
 Prob3 = 1 / (Count[modelsLengths, #] & /@ modelsLengths);
Probini = Prob1 * Prob2 * Prob3 // N;
```

Poisson Hierarchical with 3 levels;

```
A[lambda_] :=
 lambda ^ (-1 / 2) * Exp[- (lambda + 1)] * Hypergeometric0F1[0.5, lambda] / Gamma[0.5]
B[p_] := NIntegrate[lambda ^ p * Exp[-lambda] * A[lambda] / Factorial[p],
   {lambda, 0, 500}];
Prob1b = Table[B[j], {j, 1, k}] / Total[Table[B[j], {j, 1, k}]];
Prob1 = Table[Prob1b[[Length[models[[i]]]]], {i, 1, Numberofmodels}];
 Prob2 = 1 / (Numbersubgroups2[[Length /@ models]]);
 modelsLengths = Sort /@ Map[Length, models, {2}];
 Prob3 = 1 / (Count[modelsLengths, #] & /@ modelsLengths);
Probini = Prob1 * Prob2 * Prob3 // N;
```

Posterior probabilities

```
posterior1 = Probini * Marginals;
posterior = posterior1 / Total[posterior1];

result = Partition[Riffle[posterior, models], 2];
results = Sort[result, #1[[1]] > #2[[1]] &];

Mean0[x_, n_, t_] := NIntegrate[10 ^ 1000 * zeta * G[x, n, t, zeta], {zeta, 0, 1}];
Mean1[x_, n_] := Mean0[x, n, t0] / 10 ^ 1000;

Numerator1 = Parallelize[MapThread[Mean1, {xpartitions, npartitions}]];
```

```
Numerator0 = Table[Numerator1[[j]], {j, 2, Numberofmodels}];
Numeratorfirst = (xpartitions[[1]] + 1) !
    (npartitions[[1]] - xpartitions[[1]]) ! / (npartitions[[1]] + 2) ! // N;
Numerator1 = Join[Numeratorfirst, Numerator0];

zetaestimate = Numerator1 / Marginals;

H[zeta_, x_, n_] := G[x, n, t0, zeta] / Pred[x, n];
L1 =
  H[zeta, xpartitions[[#]], npartitions[[#]]] & /@ Range[Length[xpartitions]];

LikelihoodBMA = L1.posterior;
PDFBMA = ProbabilityDistribution[LikelihoodBMA, {zeta, 0, 1}];
```

Results models

```
results

Mostprobablemodel = results[[1]]

Positionhomogeneity =
 Flatten[Position[Table[results[[j]][[2]], {j, 1, Numberofmodels}], models[[1]]]]

Probhomogeneity = results[[Positionhomogeneity[[1]]]][[1]]

Positionheterogeneity = Flatten[Position[
   Table[results[[j]][[2]], {j, 1, Numberofmodels}], models[[Numberofmodels]]]]

Probheterogeneity = results[[Positionheterogeneity[[1]]]][[1]]
```

Results metaparameter BMA

```
MetaparameterBMA = zetaestimate.posterior

Plot[LikelihoodBMA,
 {zeta, 0, Min[Round[5 * MetaparameterBMA * 10] / 10, 1]}, PlotRange → All]
```
